# Governance of antimicrobial resistance in Central Africa (2015–2025): a systematic review of policies, One Health coordination and funding

**DOI:** 10.1093/jacamr/dlag093

**Published:** 2026-05-30

**Authors:** Gabriel Tchuente Kamsu, Ingrid Cécile Djuikoue, Benjamin D Thumamo Pokam, Cedric Dylan Seugnou Nana, Clement Ngandjui Yonga, Mallila Georgia Mboupaing, Marie Stella Marehin, Nicole Prisca Makaya Danguil Nieko, Flambel Sanou Kue, Tatiana Banze Mewehu, Dorine Tseuko, Siapsantos Hironi, Marie Therese Mboliyou, Rita Armelle Gatse Bouya, Teke Ruffin Apalata, Nicolas Antoine -Moussiaux

**Affiliations:** Central African Network for the Integrated Control of Antimicrobial Resistance (CANIC-AMR), Yaoundé, Cameroun; Faculty of Medicine and Health Sciences, Walter Sisulu University, Mthatha, South Africa; Central African Network for the Integrated Control of Antimicrobial Resistance (CANIC-AMR), Yaoundé, Cameroun; Department of Public Health, Université des Montagnes, Bangangté, Cameroon; Central African Network for the Integrated Control of Antimicrobial Resistance (CANIC-AMR), Yaoundé, Cameroun; Department of Medical Laboratory Science, Faculty of Health Sciences, University of Buea, Buea, Cameroon; Central African Network for the Integrated Control of Antimicrobial Resistance (CANIC-AMR), Yaoundé, Cameroun; Central African Network for the Integrated Control of Antimicrobial Resistance (CANIC-AMR), Yaoundé, Cameroun; Department of Veterinary Animal Resource Management, FARAH Research Unit, Faculty of Veterinary Medicine, University of Liège, 4000 Liège, Belgium; Central African Network for the Integrated Control of Antimicrobial Resistance (CANIC-AMR), Yaoundé, Cameroun; Central African Network for the Integrated Control of Antimicrobial Resistance (CANIC-AMR), Yaoundé, Cameroun; Department of Psychology, Université Omar Bongo de Libreville, Libreville, Gabon; Central African Network for the Integrated Control of Antimicrobial Resistance (CANIC-AMR), Yaoundé, Cameroun; National Public Health Laboratory, Université Marien NGOUABI, Brazzaville, République du Congo; Central African Network for the Integrated Control of Antimicrobial Resistance (CANIC-AMR), Yaoundé, Cameroun; Department of Public Health, Université des Montagnes, Bangangté, Cameroon; Central African Network for the Integrated Control of Antimicrobial Resistance (CANIC-AMR), Yaoundé, Cameroun; Central African Network for the Integrated Control of Antimicrobial Resistance (CANIC-AMR), Yaoundé, Cameroun; Laboratoire de Santé Publique, Yaoundé, Cameroun; Central African Network for the Integrated Control of Antimicrobial Resistance (CANIC-AMR), Yaoundé, Cameroun; Central African Network for the Integrated Control of Antimicrobial Resistance (CANIC-AMR), Yaoundé, Cameroun; Central African Network for the Integrated Control of Antimicrobial Resistance (CANIC-AMR), Yaoundé, Cameroun; Faculty of Medicine and Health Sciences, Walter Sisulu University, Mthatha, South Africa; Département des Sciences de la Santé Publique, Faculté de Médecine, Université de Liège, Liège, Belgique

## Abstract

**Background:**

Antimicrobial resistance (AMR) poses a major threat to effective treatment and global health security, with disproportionate impacts in low- and middle-income settings. In Central Africa, health system fragility, limited surveillance capacity and weak multisectoral coordination heighten vulnerability. Despite momentum following the WHO Global Action Plan, the status of AMR governance, One Health coordination and financing in the subregion remains insufficiently characterized.

**Objectives:**

To systematically assess AMR governance in Central Africa (2015–2025), focusing on policy frameworks, One Health coordination and financing mechanisms.

**Methods:**

We conducted a PRISMA 2020-compliant systematic review registered in PROSPERO (CRD420251082393). Six databases were searched, along with grey literature, including national policy documents, institutional reports and consultations with AMR focal points. Eligible sources addressed AMR policy, coordination, surveillance and funding.

**Results:**

Of the 4464 records identified, 27 documents were included (19 peer-reviewed articles, one report, seven national action plans). Evidence was concentrated in Cameroon, with limited data from other countries. National action plans aligned with global standards were identified in six countries, while none were available for Equatorial Guinea or the Republic of Congo. Across the region, governance was characterized by partial policy alignment but weak implementation, limited operationalization of One Health, fragmented surveillance and reliance on external funding. Key constraints included inadequate laboratory capacity, weak stewardship systems and insufficient domestic financing.

**Conclusion:**

AMR governance in Central Africa has progressed at the policy level but remains operationally constrained. Bridging the gap between policy and implementation requires strengthened institutionalization of One Health, improved accountability and sustainable domestic investment.

## Introduction

Antimicrobial resistance (AMR) is a major threat to global public health in the 21^st^ century.^1^ It undermines effective treatment, increases morbidity and mortality and imposes substantial economic costs, especially in low- and middle-income countries (LMICs).^2^ Rising multidrug resistance (MDR) is associated with treatment failure, longer hospital stays and wider health inequalities.^3^ In order to curb this threat, the World Health Organization (WHO) launched the Global Action Plan (2015 and reaffirmed in 2019) framework calling on member states to develop national action plans (NAP) guided by a multisectoral One Health approach.^4–6^ One Health recognizes the interdependence of human, animal and environmental health and requires coordinated action across sectors to reduce AMR.^7^ In this context, AMR governance encompasses the political, institutional, regulatory and financial mechanisms that guide and oversee multisectoral responses.^8,9^ Effective governance relies on coherent policies, integrated surveillance, clear accountability and sustainable financing.

Central Africa faces persistent challenges in translating policy intent into implementation. Available evidence indicates a high AMR burden, driven by inappropriate antimicrobial use in human, veterinary and agricultural settings and compounded by weak surveillance.^10,11^ Governance capacity also varies widely across the subregion. Many countries lack operational regulatory frameworks, functional surveillance systems and durable institutional arrangements for coordination.^12,13^ Limited infrastructure, constrained financing and underdeveloped One Health coordination further restrict delivery. Although international commitments are increasingly visible, implementation remains fragmented.

The rationale for focusing specifically on Central Africa lies in its unique convergence of structural vulnerabilities, including fragile health systems, recurrent humanitarian crises and limited institutional capacity, which distinguish it from other African subregions where AMR governance structures are comparatively more advanced.^14^ Prior to 2015, AMR governance in the subregion was largely underdeveloped or absent in several countries; where efforts did exist, but were often confined to tertiary healthcare settings and implemented without comprehensive national policy frameworks. These early initiatives were characterized by limited policy coordination, weak and fragmented surveillance systems and minimal integration of multisectoral approaches, thereby providing an important baseline for understanding subsequent developments. In addition, the subregion remains underrepresented in the global AMR literature, with existing evidence often fragmented, country-specific or heavily concentrated in a few settings, such as Cameroon, thereby limiting comprehensive regional understanding. Countries in Central Africa, including Cameroon, Democratic Republic of Congo (DRC), Gabon, Chad, Central African Republic (CAR), São Tomé and Príncipe, Equatorial Guinea and Republic of Congo, exhibit heterogeneous governance trajectories, ranging from relatively well-established policy frameworks to contexts where national strategies are absent or poorly documented. This variability underscores the need for a systematic and comparative assessment of governance structures across the subregion.

Furthermore, while global and regional analyses of AMR governance have been conducted, they often aggregate Central Africa within broader African groupings, obscuring context-specific challenges related to One Health coordination, surveillance capacity and financing constraints. As a result, critical gaps persist in understanding how governance frameworks are designed, implemented and operationalized in this specific geopolitical and health system context.

Evidence on governance effectiveness is limited, and no comprehensive subregional assessment has been synthesized. Accordingly, this study aims to systematically assess AMR governance in Central Africa between 2015 and 2025, with a focus on policy frameworks, One Health coordination mechanisms, surveillance systems and financing structures. Specifically, this review seeks to answer the following research question: to what extent have Central African countries developed and operationalized effective AMR governance systems in alignment with the One Health approach since the adoption of the Global Action Plan?

## Methods

### Protocol registration and eligibility criteria

This scoping review followed PRISMA 2020^15^ and was registered in PROSPERO (CRD420251082393). It examined AMR governance within a One Health framework in eight Central African countries (Cameroon, CAR, Chad, Republic of Congo, DRC, Equatorial Guinea, Gabon and São Tomé and Príncipe) from 2015 to 2025. We included two evidence types to capture governance arrangements: (i) empirical studies using quantitative, qualitative, or mixed methods that reported on AMR policies, governance structures or implementation processes and (ii) official national documents, including NAP-AMR, One Health strategic frameworks and government implementation or activity reports that reported on coordination, surveillance and monitoring and financing. Eligible empirical study designs included cross-sectional studies, qualitative policy analyses, implementation research and mixed-methods evaluations addressing AMR governance or One Health coordination, as well as grey literature such as national policy documents, institutional reports and consultations. We excluded editorials, opinion pieces and commentaries without original data or substantive policy content. Only documents published in English, Portuguese, Spanish and French were included, reflecting the subregion’s predominant official and scientific languages were eligible.

This study assessed governance domains covering national policies and action plans, multisectoral coordination platforms, surveillance and monitoring systems, capacity-building initiatives and financing mechanisms. A governance mechanism was considered present when at least one formally documented instrument or structure was identified (e.g. an approved NAP-AMR, an operational One Health platform, a budget line or a monitoring and evaluation framework). Primary outcomes were the existence, design and implementation status as reported in included sources. Implementation status was defined based on reported evidence of operationalization, including functionality of coordination platforms, execution of planned activities or availability of monitoring data. Secondary outcomes included reported barriers and enabling factors, alignment with WHO, Food and Agriculture Organization of the United Nations (FAO) and World Organization for Animal Health (WOAH, formerly OIE) guidance and evidence on surveillance and response capacity. Additional outcomes of interest included the extent of multisectoral integration under the One Health approach, the presence of legal and regulatory frameworks and the balance between domestic and external financing supporting AMR activities.

### Information sources and search strategy

A comprehensive literature search was conducted in MEDLINE (via PubMed), Scopus, Web of Science Core Collection, Cochrane Library, WHO Global Index Medicus and African Journals Online. Searches were restricted to materials published between January 2015 and 31 May 2025. The strategy accounted for the official languages of the jurisdictions under study (English, French, Portuguese and Spanish) to minimize language-related selection bias. Where key national documents were not available in English, AMR focal points in participating countries supported translation of selected documents to facilitate data extraction and cross-country comparison.

The search strategy was developed in collaboration with an information specialist and reported in line with PRISMA 2020 recommendations. The core search concepts included: (‘antimicrobial resistance’ OR ‘AMR’) AND (‘governance’ OR ‘policy’ OR ‘legislation’ OR ‘health policy’) AND (‘One Health’ OR ‘multisectoral’ OR ‘intersectoral’) AND (‘Central Africa’ OR ‘Cameroon OR ‘Democratic Republic of the Congo’ OR ‘Chad’ OR ‘Gabon’ OR ‘Republic of the Congo’ OR ‘Central African Republic’ OR ‘Equatorial Guinea’ OR ‘São Tomé and Príncipe’). It combined free-text keywords with controlled vocabulary terms, adapted to the thesaurus and syntax of each database using appropriate field tags and indexing systems. Complete database-specific search strategies, including full Boolean logic, truncations, proximity operators where applicable and controlled vocabulary mappings, are provided in the supplementary materials (available as Supplementary data at *JAC-AMR* Online) to support reproducibility. Database searches were conducted independently by C.D.S.N. and F.S.K. across the selected databases.

To capture policy-relevant evidence not indexed in bibliographic databases, grey literature (national policy documents, institutional reports and consultations) was searched systematically by M.S.M. using the official websites of the WHO, FAO, WOAH, Africa Centres for Disease Control and Prevention (Africa CDC) and the United Nations Environment Programme. National government portals were also searched to identify NAP-AMR, One Health strategies and implementation or activity reports relevant to AMR governance. Reference lists of included sources were screened to identify additional eligible documents.

### Data management and selection process

**Figure 1.**
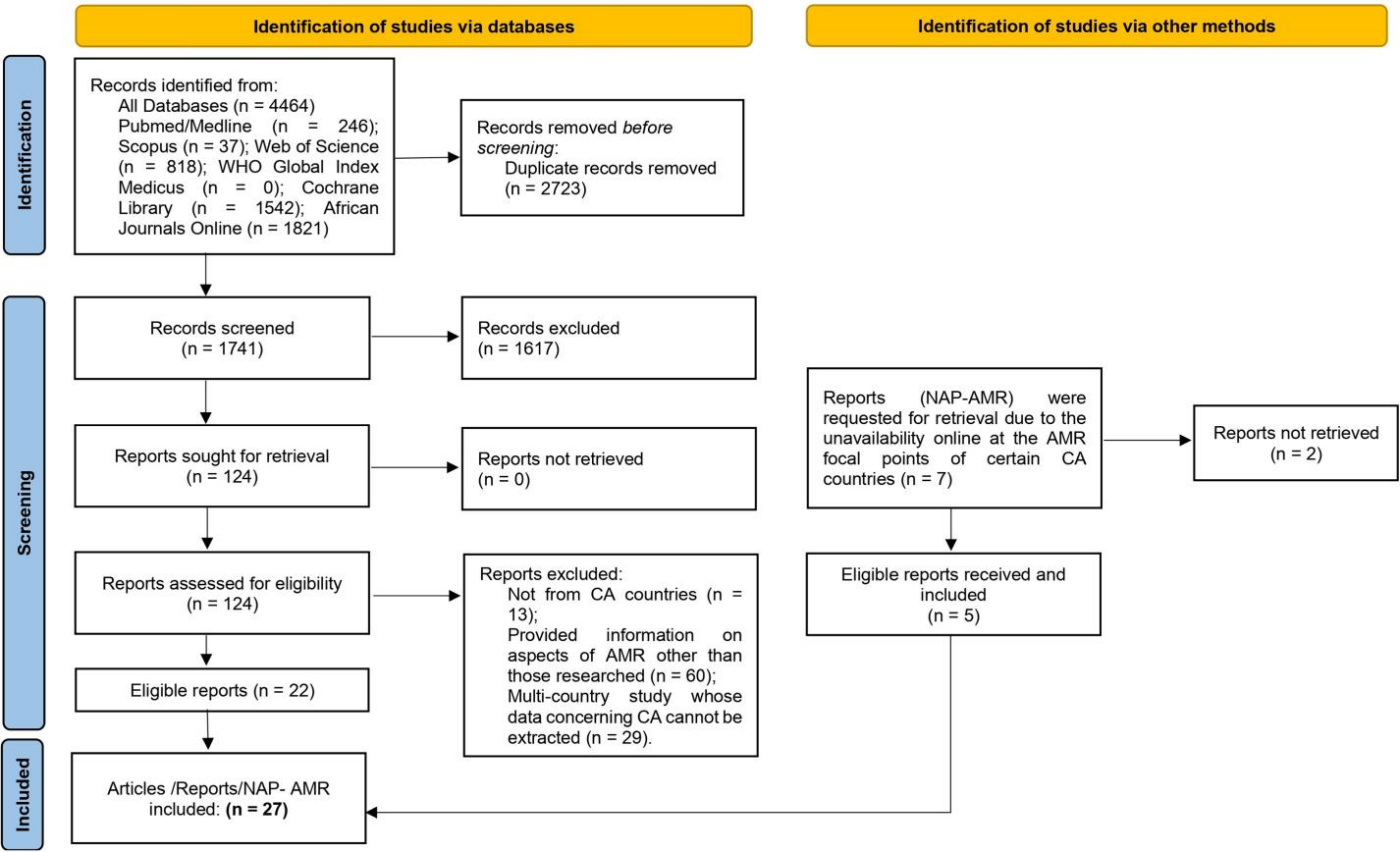
PRISMA 2020 flow diagram for new systematic reviews with included studies. n: indicate the number of records identified at each stage of the screening process.

All records retrieved from database searches were exported to EndNote for de-duplication. After de-duplication, records were uploaded to Rayyan for screening.^16^ Two reviewers (G.T.K. and I.C.D.) independently screened titles and abstracts and then independently assessed full texts of potentially eligible records. Eligibility criteria were applied at both stages, and reasons for full-text exclusion were documented. Where multiple reports described the same study, reports were linked and the report providing the most complete information relevant to the review outcomes was retained. Disagreements were resolved through discussion, with adjudication by third reviewers (T.R.A. and B.D.T.P.). Prior to formal screening, a calibration exercise was conducted on a subset of records to ensure consistent application of eligibility criteria. Inter-reviewer agreement was assessed at both the title/abstract and full-text screening stages, with discrepancies resolved through consensus to maintain methodological rigour. The selection process is presented in the PRISMA 2020 flow diagram (Figure 1).

### Data extraction

Data extraction was performed independently by C.N.Y. and M.G.M. using a standardized, pilot-tested extraction form. Extracted items included bibliographic and document characteristics (first author or issuing body, year, country), features of AMR governance frameworks (policy instrument type, One Health coordination arrangements and financing sources and modalities), and reported information on NAP implementation, including progress, barriers and enabling factors. Disagreements were resolved through discussion, with adjudication by senior third reviewers (B.D.T.P. and N.A.M.).

### Risk of bias and certainty assessment

Empirical studies (qualitative, quantitative or mixed methods) were appraised using the Mixed Methods Appraisal Tool.^17^ Design appropriateness, data collection and analysis and coherence between research questions, methods and conclusions were assessed by G.T.K. and I.C.D.

Policy and governance documents (including NAP-AMR, One Health strategies and official reports) were assessed using a structured alignment approach. Documents were examined against the core components of the WHO Global Action Plan on AMR to determine whether key governance requirements were addressed, including institutional arrangements, multisectoral coordination, surveillance and monitoring and financing provisions.

Certainty of the synthesis was interpreted in light of these appraisals. Formal grading approaches such as GRADE were not applied because a substantial proportion of the evidence consisted of governance instruments and policy documents. Instead, appraisal outputs were used to contextualize findings, identify limitations and reduce the risk of overinterpretation where governance arrangements were weakly specified or poorly documented.

### Data synthesis

We conducted a framework-based qualitative synthesis of all included sources and summarized findings in a comparative table. Data were charted against a predefined analytic framework aligned with the review objectives. The framework comprised three domains: national policy frameworks, multisectoral One Health coordination mechanisms (including surveillance and monitoring arrangements) and financing strategies. The synthesis followed an iterative process involving data familiarization, open coding of extracted data and subsequent grouping of codes into thematic categories within each domain. Themes were identified inductively and refined through constant comparison across studies and countries to ensure consistency and analytical depth. Coding and theme development were performed independently by two reviewers, who then discussed their findings to reach consensus, thereby enhancing the credibility of the synthesis. Patterns were compared across countries to identify common approaches, gaps and context-specific features. Reporting was in accordance with PRISMA 2020.^15^

## Results

### Study characteristics

The database search identified 4464 records. After removing 2723 duplicates, 1741 unique records were screened by title and abstract and 1617 were excluded. Overall, 124 full-text records were assessed. Of these, 102 were excluded because they did not originate from Central Africa (*n* = 60) or did not address AMR governance or policy (*n* = 42) (Figure 1). An additional five records were identified through other sources (*n* = 5), including direct contact with national AMR focal points to obtain NAP-AMR documents that were not publicly accessible. Of seven countries contacted, five provided documents. Equatorial Guinea and the Republic of Congo had no national AMR plan at the time of the review.

The final synthesis included 27 documents (Table S1): 19 peer-reviewed articles, one activity report and seven NAP-AMR. Document production was concentrated in Cameroon (12 articles, one report and two plans for 2021–2024 and 2024–2028), followed by the DRC (four articles and one plan) and Gabon (three articles and one plan). The Central African Republic, Chad and São Tomé and Príncipe contributed NAPs but no peer-reviewed articles.

Scientific articles focused on laboratory-based AMR surveillance, antimicrobial use in livestock production, healthcare practices and implementation constraints in resource-limited or conflict-affected settings. National reports and action plans published since 2018 placed greater emphasis on policy strengthening and multisectoral coordination within a One Health approach. Across the included national plans, time horizons clustered from 2021 onwards, reflecting a shift from problem description towards formalized strategies and implementation planning.

### National AMR governance structures, including action plans, policies and regulatory frameworks

NAPs are the primary policy instrument for AMR control in Central Africa.^18,19^ Cameroon, Gabon, São Tomé and Príncipe, CAR, the DRC and Chad developed NAPs aligned with the WHO Global Action Plan (Table 1). These plans prioritize coordination, antimicrobial use regulation and surveillance strengthening. Equatorial Guinea and the Republic of Congo were not yet engaged in this cycle at the time of review. Across countries, recurrent constraints included incomplete regulatory frameworks, limited resources, weak coordination and underdeveloped monitoring and evaluation systems.^20–26^

**Table 1.** AMR governance in Central Africa: national plans and gaps

Country	National plan/Strategy	Coordination and institutional framework	Strengths	Weaknesses/Limitations
Cameroon	NAP-AMR adopted in 2018, revised in 2023 → NAP 2024–2028	Coordinated by the National Public Health Laboratory (NPHL, Ministry of Public Health)	Multisectoral approach, WHO alignment, 2023 revision, strengthening the legal framework	An incomplete regulatory framework, unclear institutional responsibilities, no dedicated budget and pharmaceutical directives limited to outside hospitals
Gabon	NAP 2019–2023, new NAP 2025–2029	Multisectoral governance (human, animal, agricultural, environmental health)	Stakeholder mobilization, One Health integration and planned strengthening of lab capacities	Lack of operational surveillance, weak regulatory framework (certification, accreditation, quality control)
CAR	NAP validated in 2023 (aligned with the WHO 2015 plan)	Creation of a national executive secretariat for coordination	Focus on pharmaceutical regulation, curriculum reform and nosocomial infection control programmes	The regulatory framework is still embryonic and needs legal consolidation
DRC	AMR was integrated into the National Health Development Plan (2015) and NAP-AMR (2018)	Ministry of Health with the One Health approach	Strong political will, WHO alignment	No formal regulatory framework for the veterinary/agricultural sectors, weak pharmaceutical policy, not harmonized with international standards
Chad	NAP supported by the National AMR Committee and subcommittees	AMR National Committee, Ministry of Agriculture, Directorate for Plant Protection and Packaging (DPVC) is involved	One Health approach, existing regulations on antimicrobial sales/use	Weak enforcement of regulations, lack of effective monitoring mechanisms
São Tomé and Príncipe	NAP 2023–2025	Led by Ministries of Health/Education with national support	Focus on pharmaceutical regulation, curriculum reform and nosocomial infection control programmes	Regulatory framework is still embryonic, needs legal consolidation

NPHL, National Public Health Laboratory; CAR, Central African Republic; WHO, World Health Organization; DRC, Democratic Republic of the Congo; NAP-AMR, National Action Plan on Antimicrobial Resistance; NAP, National Action Plan.

Implementation capacity varied across the subregion. Cameroon and Gabon showed more advanced institutional arrangements and laboratory capacity-building initiatives, but enforcement and routine coordination remained limited.^21,22,27–29^ The Central African Republic and Chad focused on establishing national coordination bodies, yet implementation capacity remained constrained.^23,26^ São Tomé and Príncipe advanced regulatory reforms, including the creation of a national medicines regulatory authority.^24^ In the DRC and the Republic of Congo, governance was further hindered by limited enforceable legislation on antimicrobial use in veterinary and agricultural sectors, despite political recognition of AMR as a priority and, in some settings, formal planning.^19,25,30,31^

Overall, countries are converging towards more integrated AMR governance, but institutional capacity remains uneven. Gaps most consistently affected coordination operationalization, regulatory consolidation and sustainable resource mobilization in line with international standards.^29,32,33^

### Multisectoral coordination for AMR: One Health governance arrangements

Across Central Africa, AMR coordination is commonly framed through a One Health approach linking human, animal and environmental sectors.^32,34^ Countries differ in how coordination is formalized, how consistently non-human sectors participate and how operational arrangements are maintained. In most settings, coordination is described through multisectoral platforms but mandates and routine functionality vary.

In Cameroon, the NAP-AMR involves seven ministries and multiple partners and is coordinated through a multisectoral committee, while the One Health platform has remained only partially formalized since its establishment in 2012.^20,21,32^ In Chad, a multisectoral group spanning human, animal and plant sectors was formalized in 2021, but institutional coverage remains incomplete in the absence of a dedicated food and environmental safety agency.^26^ In the DRC, coordination involves multiple ministries and the WHO, yet documents report limited institutionalization and weak intersectoral collaboration.^25,33^ In the Central African Republic, a national executive secretariat coordinates multiple ministries and high-level offices, with partner support and documents describe provisions for communication and resource mobilization.^23^ In Gabon, the 2025–2029 plan assigns leadership to the Ministry of Health and describes joint committees and technical groups supporting market surveillance and linkages between surveillance networks.^22^ In São Tomé and Príncipe, a multisectoral team involving key ministries coordinates actions and reports laboratory strengthening and workforce training, supported by the WHO and national media activities.^24^ In the Republic of Congo, documents report multisectoral coordination aligned with One Health principles, despite the absence of a publicly available NAP at the time of review.

Overall, the subregion shows broad adoption of One Health coordination, but with uneven institutional maturity. Common constraints relate to incomplete formalization, variable inclusion of non-human sectors and limited operational capacity to sustain routine coordination.

#### AMR surveillance in Central Africa

AMR surveillance in Central Africa is commonly framed against WHO guidance and Global AMR Surveillance System (GLASS) standardization (Table 2),^35–37^ but implementation remains uneven, particularly for integrated antimicrobial use and consumption monitoring. In Cameroon, an integrated sentinel system became operational in 2021 (17 sites across six regions) with standard operating procedures for human and animal health.^18,21^ Data are centralized at the National Public Health Laboratory (LNSP) and reported to the Directorate for Emerging and Pandemic Diseases (DLMEP), yet routine multisectoral collection of antimicrobial use data is not described. In Chad, surveillance is guided by a strategic plan with sentinel sites and laboratories across five provinces, supported by a national guide awaiting validation.^26^ In the DRC, sources report no active national system for AMR and antimicrobial consumption, with monitoring largely restricted to selected pathogens such as those linked to tuberculosis and typhoid fever.^19,25,33,38^ In Gabon, the 2025–2029 plan proposes strengthening surveillance and research through a laboratory network for regular national assessments, although sources describe limited sentinel coverage and uneven laboratory access despite pharmacovigilance participation since 2022.^22^

**Table 2.** Surveillance and financing of AMR governance in Central Africa

Country	Coordination mechanism (One health)	surveillance system	Financing
Cameroon	Multisectoral Coordination Committee (MCC) involving 7 ministries + technical partners; supported by MTaPS/USAID and technical working groups	Surveillance is partially functional; weak representation of the environmental sector	Mixed sources: international partners (USAID/MTaPS, GHSC-PSM, WHO, OCEAC, EU, FAO/OIE, METABIOTA, Georgetown), national funds (State Budget, BIP, PRODEL, registration fees)
Gabon	Ministries of Health, Agriculture and Environment engaged in the One Health framework	No integrated AMR surveillance system; limited epidemiological reporting	Limited resources, dependent on external aid (WHO)
CAR	National Executive Secretariat involving Health, Agriculture, Environment, Economy, Presidency, Prime Minister’s office	Weak surveillance systems	Supported by the World Bank, REDISSE, WHO
São Tomé and Príncipe	Multisectoral group: Health, Agriculture, Environment, Infrastructures + hospitals, professional bodies, media, WHO	Surveillance of pharmaceuticals in aquatic environments; cross-training of health professionals	National resources + international partner (WHO)
DRC	Ministries of Health, Fisheries & Livestock, Agriculture, Environment, Higher Education, Research + WHO	Surveillance plans remain theoretical; diagnostic labs are weak; customs/regulatory collaboration is limited	WHO
Chad	National AMR Committee with regular meetings; focal point supported financially; One Health integration (human, animal, plant health)	Partial integrated surveillance	National budget + WHO

AMR, Antimicrobial Resistance; MTaPS/USAID, Medicines, Technologies and Pharmaceutical Services Programme/United States Agency for International Development; USAID/MTaPS, United States Agency for International Development/Medicines, Technologies and Pharmaceutical Services; GHSC-PSM, Global Health Supply Chain Programme—Procurement and Supply Management; WHO, World Health Organization; OCEAC, Organization for Coordination in the Fight Against Endemic Diseases in Central Africa; EU, European Union; FAO/OIE, Food and Agriculture Organization of the United Nations/World Organization for Animal Health; BIP, Public Investment Budget; PRODEL, Livestock Development Programme; CAR, Central African Republic; REDISSE, Regional Disease Surveillance Systems Enhancement Programme; DRC, Democratic Republic of the Congo; AMR, Antimicrobial Resistance.

São Tomé and Príncipe proposes an integrated One Health model covering animal health surveillance, antimicrobial distribution and use and food-related monitoring, with data integration into the national health information system (e-SIS).^24^ In the Republic of Congo, eight sentinel sites were established in 2024 using standardized procedures and digital tools, but sources describe passive surveillance and the absence of an active national system and central database [Table 2].

Overall, reported constraints relate to laboratory capacity, limited inclusion of non-human sectors and weak integration of AMR and antimicrobial use data across systems.^18,22,23,26,38^

#### Financing AMR governance in Central Africa

Across countries, funding for AMR governance was reported as a mix of partner support and domestic resources, with limited evidence of dedicated budget lines and variable costing of NAP implementation (Table 2). International partners frequently cited across the subregion included United States Agency for International Development (USAID)-supported programmes, WHO, the World Bank Group and Regional Disease Surveillance Systems Enhancement (REDISSE), the European Union (EU), FAO and WOAH, and other technical partners. Domestic contributions were reported through state budgets and, in some settings, fees and institutional support. Common constraints related to funding predictability, limited domestic resource mobilization and shortages in skilled personnel needed to sustain multisectoral coordination.^32,39^

In Cameroon, sources reported no dedicated budget line for AMR activities, with several interventions financed through technical and financial partners.^21^ Successive NAPs included costed activities, yet funding continuity was described as vulnerable to partner transitions, including the withdrawal of Metabiota and reported uncertainty linked to changes in US support. In Chad, the first strategic plan was described as underfunded, and the cost of the second 4-year plan was estimated at USD 2 370 172.67, alongside ongoing efforts to mobilize financing.^26^

In the CAR, the NAP-AMR identified limited material, financial and human resources as a major implementation risk. The roadmap included cost estimation, budgeting and partner roundtables intended to support resource mobilization and more sustainable financing.^23^ In Gabon, the 2025–2029 plan reported limited financial resources as a key constraint and estimated total implementation costs at USD 28 516 732.49 over five years. The plan positioned government as the implementation lead, with support anticipated from technical partners, foundations, Non-Governmental Organizations, private sector actors and communities. High-level endorsement was documented through adoption by the Council of Ministers.^22^

In São Tomé and Príncipe, the plan described WHO-supported financing and technical assistance, complemented by partner involvement including civil society. Total costs were estimated at USD 1 333 100, and advocacy activities were outlined to institutionalize the national medicines regulatory authority. Sources also highlighted dependency on external support and limited domestic mobilization as constraints on sustainability.^24^ In the Republic of Congo, sources described AMR-related projects and awareness activities, but no dedicated budget for NAP implementation was reported, and funding was described as insufficient to operationalize coordination mechanisms and surveillance systems.

Overall, the subregional evidence indicates that financial viability depends on sustained mobilization of domestic and partner resources, with persistent financing gaps continuing to limit implementation of AMR governance priorities.^12^

### Implementation quality and compliance with international standards

The quality of the implementation of national plans to combat AMR in Central Africa reveals mixed progress and major challenges affecting compliance with international standards (Table 3).

**Table 3.** AMR implementation and compliance in Central Africa

Country	Implementation quality	Compliance with international standards	Main limitations
Cameroon	Partial implementation despite strategic alignment with WHO/OIE/FAO	Data completeness is high, but limited interoperability, weak digital platforms, partial adherence to GLASS and uneven application of therapeutic guidelines	No integrated real-time system; gaps in veterinary and environmental data; weak pharmacovigilance; incomplete regulation of antimicrobial sales; weak lab quality (SLIPTA, AMR Scorecard)
Gabon	Political will is present but limited in implementation	Lack of local prescribing guidelines; poor training programmes; weak health information system; absence of quality assurance mechanisms	Few clinical microbiology labs, unreliable strategic documents, no national prescription directives and limited infection prevention education
CAR	The operational plan includes a structured monitoring and evaluation framework	Partial compliance due to weak labs, poor infrastructure and a lack of data system standardization.	Limited laboratory capacity, a weak healthcare system and a lack of consolidated national data.
São Tomé and Príncipe	Structured monitoring and evaluation in place; voluntary commitments to reduce critical antimicrobial use	Shows a strong willingness to align with international standards, with planned periodic evaluations	Results have not yet been documented; there is a need for stronger evidence of implementation.
DRC	Implementation is constrained by low microbiological surveillance and weak diagnostic capacity.	Awareness campaigns and training aligned with international standards; priorities identified (lab strengthening, sentinel networks, international integration)	There are very few reference labs, a lack of standardized protocols and quality testing and no national monitoring and evaluation system.
Chad	Tangible organizational support, regular coordination of activities and incentives for the national focal point.	Weak compliance due to informal drug distribution circuits, poor enforcement of laws and counterfeit medicines.	Antibiotics sold without prescription; illegal vendors; weak lab data undermining policy design.

WHO/OIE/FAO, World Health Organization/World Organization for Animal Health/Food and Agriculture Organization of the United Nations; GLASS, Global Antimicrobial Resistance and Use Surveillance System; SLIPTA, Stepwise Laboratory Improvement Process Towards Accreditation; AMR, antimicrobial resistance; CAR, Central African Republic; DRC, Democratic Republic of the Congo.

#### Quality of implementation of the NAP against AMR in Central Africa

The implementation of NAPs to combat AMR in Central Africa has shown uneven progress, constrained by organizational, technical and infrastructural limitations. In Cameroon, despite the existence of a strategic framework aligned with the guidelines of the WHO, OIE and FAO, implementation remains partial. The absence of an integrated, interoperable and real-time system restricts data-driven analysis and response, particularly in the veterinary and environmental sectors.^18,29,36,40^ A 2021 evaluation highlighted strong data usefulness (88.9%) and satisfactory completeness of reporting (98.9%) but weak simplicity (64.3%), stability (58.6%) and acceptability (58.6%), with an overall data quality rating of 11.05%.^18^ These shortcomings are linked to the recent implementation of the system, a low training rate among actors (50%), the limited number of laboratories and the absence of standardized technical platforms.^18,41^ Passive surveillance and the lack of integration within the environmental sector further limit the operational relevance of the data.^18^

In Gabon, the absence of national guidelines for rational antibiotic use, insufficient continuous training and lack of operational clinical microbiology laboratories constrain effective implementation.^22,28,41^ The quality assurance systems and mechanisms used to regulate professional practices remain weak and are compounded by a lack of coordination and synergy among stakeholders.^22^ In the CAR, the operational plan includes a structured monitoring and evaluation framework, but implementation remains partial due to limited laboratory capacity, weak health infrastructure and the absence of a consolidated data management system.^12,23^

In São Tomé and Príncipe, a structured monitoring and evaluation system has been established, alongside voluntary commitments to reduce critical antimicrobial use, strengthen institutional and technical capacity in hospital and veterinary laboratories, provide training for laboratory technicians and develop standard operating procedures (SOPs) for microbiology.^24^ However, limited technological resources and diagnostic capacity continue to hinder the effectiveness of implementation. In the DRC, microbiological surveillance coverage is limited, reference laboratories are nearly absent and standardized protocols are poorly developed, which complicates the comparability of data across laboratories.^19,33,38^ Most testing remains therapeutic in scope, and few laboratories are formally accredited. Training and awareness initiatives are underway, but the absence of national monitoring and evaluation systems restricts the adaptation of interventions to local realities.

In Chad, logistical and institutional support is tangible, and regular coordination mechanisms function relatively well. However, poor adherence to standardized procedures for antimicrobial susceptibility testing, insufficient prevention groups and a lack of reagents and culture media compromise the quality of implementation.^26^ Finally, in the Republic of Congo, implementation has been marked by strong momentum in strengthening surveillance and coordination, with eight sentinel sites and the digitalization of monitoring tools. Nevertheless, systemic, regulatory, clinical and infrastructural weaknesses persist, whereas the absence of a formal institutional framework undermines national governance and the operational quality of the NAP-AMR.

#### The NAP’s compliance with international standards in Central Africa

Compliance with international standards in AMR control across Central Africa remains uneven and hampered by significant structural and regulatory challenges. In Cameroon, regulatory integration remains limited, particularly in areas such as pharmacovigilance, sectoral accountability and antimicrobial sales regulation, largely due to the absence of a robust national strategy for consumption monitoring.^36,40^ Laboratory assessments using Stepwise Laboratory Improvement Process Towards Accreditation (SLIPTA) and AMR scorecard standards reveal technical deficiencies, a lack of quality assurance mechanisms and only partial adherence to GLASS requirements.^36,42^ The 2017 Joint External Evaluation assigned Cameroon a score of 1/5 across all AMR-related components, underscoring weak compliance.^32^ However, the 2024–2028 NAP-AMR incorporates International Health Regulations^43^ indicators, and the country has adopted the WHONET platform for surveillance data entry.^18^ The Codex Alimentarius is also referenced as a quality assurance standard for food products.^21^

In Gabon, the absence of national guidelines for rational antibiotic use and the lack of functional quality assurance mechanisms significantly limit alignment with international standards.^22,28^ In the CAR, limited laboratory capacity, the absence of standardization and the lack of a consolidated data management system hinder compliance with global standards.^12,23^ Nonetheless, the country aims for progressive enrolment in WHO’s -GLASS, and its national plan references the Codex Alimentarius in relation to foodborne AMR guidelines.^23^

In São Tomé and Príncipe, efforts are underway to align the national framework with international standards through the establishment of a structured monitoring and evaluation system, planning of periodic assessments and development of SOPs for antimicrobial management.^24^ The Codex Alimentarius is also cited as a reference for food safety and quality assurance. In the DRC, the lack of standardized protocols and regular quality testing reduces data reliability and undermines adherence to international standards.^19^ Strengthening laboratory capacities, establishing sentinel surveillance networks and integrating into global monitoring platforms are identified as key priorities to improve alignment.^19,38^ However, the absence of a national monitoring and evaluation system remains a major barrier.^38^

In Chad, compliance is severely constrained by the persistence of informal distribution channels, the over-the-counter sale of antibiotics without a prescription, the presence of illegal vendors and the circulation of counterfeit medicines, all of which pose significant challenges to regulatory enforcement.^26^ In addition, weak laboratory data further limit the reliability of compliance assessments and adherence to best practices.^12^ Finally, in the Republic of Congo, the existence of a national laboratory directorate and the country’s participation in the GLASS-AMR platform, as well as in the 2023 WHO Joint External Evaluation, reflect a clear commitment to aligning with international standards, although operational implementation remains hindered by structural deficiencies.^44^

### Reported barriers and enabling practices for AMR governance in Central Africa

#### Reported barriers

Across included sources, barriers clustered into four domains: data and surveillance performance, laboratory and workforce capacity, antimicrobial use practices and governance and regulatory constraints. Evidence on AMR burden and impact was described as limited and heterogeneous in sub-Saharan Africa, including Cameroon and Gabon, and WHO reporting highlighted large gaps in reliable AMR data across the African Region.^41,45,46^ Sources linked these gaps to weak surveillance systems, limited methodological standardization and constrained data sharing.^47^

Surveillance coverage and multisectoral representation were reported to be uneven. In Cameroon, data were described as concentrated in four regions, with limited reporting from animal and environmental sectors.^27,48^ Sources also noted variable data quality and limited laboratory coverage, alongside surveillance attributes reported as low in simplicity, stability and acceptability.^18,41^ In Gabon, sources described absent or insufficient sentinel sites.^22,41^ In the DRC, documents reported limited laboratory capacity, ageing infrastructure, weak protocol standardization and narrow surveillance coverage.^19^

Resource and capability constraints were repeatedly reported. Sources described shortages of functional laboratories, reagents and trained personnel, which limited routine surveillance and response activities.^29,49^ Limited molecular diagnostic capacity was also reported as a constraint on characterizing AMR dissemination.^50^

Sources further described widespread inappropriate antimicrobial use, including self-medication, inappropriate prescribing, limited diagnostic capacity and suboptimal hygiene practices in facilities and communities.^32,51–53^ At system level, documents reported weak or incomplete governance arrangements, unclear leadership and coordination challenges that hindered implementation of national plans.^21,26,32^ Additional constraints included the circulation of substandard and counterfeit medicines, poverty, geographical isolation in rural areas and pressures from emerging infectious diseases.^26,27,33,36,54^ In the Republic of Congo, sources described gaps in laboratory testing capacity, the absence of a central AMR and antimicrobial use database and limited stewardship, training and routine surveillance functions.

#### Reported enabling practices

Included sources also described enabling practices, although reporting varied by country. Implemented or operationalized measures included the formalization of technical working groups with defined mandates, laboratory strengthening through quality assurance activities and efforts to link antimicrobial consumption and AMR data to decision-making, as reported in Cameroon.^32,36,42^ Sources also described the adoption of therapeutic guidelines, although implementation was reported as uneven.^20,21^ Several documents highlighted broader stakeholder participation, including civil society and private sector engagement, as supporting ownership and continuity of interventions.^32^ Coordination across policymakers, professionals and communities was described as facilitating joint planning and resource mobilization.^27,29,51^

Across plans and reports, proposed enabling measures prioritized strengthening surveillance and laboratory systems, including standardized methodologies, timely feedback to actors and clearer reference laboratory functions.^19,28^ Documents also outlined quality assurance systems and external performance evaluation, which were inconsistently available across countries.^19,25,38^ Several sources proposed operational research initiatives, including genomic approaches for priority pathogens where capacity allowed.^23,25^

Education and risk communication were consistently described as enabling components. Included sources reported communication campaigns, continuing professional training on appropriate prescribing and hygiene practices and integration of AMR content into training curricula.^21,22,24,25,26,28^ Sources also highlighted targeted awareness activities for farmers and livestock producers to support prudent antimicrobial use.^26,50^ National plans further described intended measures in regulation, infection prevention and control, stewardship and medicine quality oversight as part of broader governance strengthening.

### Stakeholders involved in AMR governance in Central Africa: roles and levels of engagement

Across included sources, stakeholder engagement in AMR governance was described at national, regional and international levels within a One Health framing. At national level, ministries responsible for health, livestock and environment were identified as lead institutions, working through multisectoral committees to coordinate AMR activities and, where available, NAPs. Reference laboratories were reported as key technical actors supporting bacteriology and surveillance functions, including, for example, the Pasteur Centre in Cameroon, the National Institute of Biomedical Research (INRB) in the DRC and the Pasteur Institute in Bangui (Central African Republic). Academic institutions were described as contributing to workforce training and data generation.

At regional level, sources identified the Organization for the Coordination of the Fight Against Endemics in Central Africa (OCEAC) as supporting cross-border coordination and strategy harmonization.^55^ Africa CDC involvement was also referenced, including collaboration with the African Union and UN agencies in activities related to surveillance systems, laboratory capacity and workforce training.^56^ Research partnerships were reported to generate antimicrobial use and resistance data in selected settings. For example, the Mapping AMR and Antimicrobial Use Partnership, coordinated by the One Health Trust and funded by the Fleming Fund, was described in relation to work in Cameroon and Gabon.^57^

At international level, included documents described WHO, FAO and WOAH as providing normative guidance and technical support. Donor-funded mechanisms and bilateral agencies were also identified as supporting implementation through health system activities, diagnostic access and rational use initiatives.^58^ Non-governmental organizations were reported as supporting service delivery in fragile settings. Médecins Sans Frontières, for instance, was described as providing access to clinical microbiology services in parts of the DRC, the CAR and Cameroon through a combination of in-house and external laboratory arrangements.^59^

## Discussion

Our synthesis demonstrates substantial heterogeneity in AMR governance across Central Africa, encompassing strategic planning, legal frameworks, multisectoral coordination, surveillance systems and financing mechanisms. Countries with established NAPs exhibit clearer strategic intent; however, implementation remains inconsistent, under-resourced and weakly institutionalized. At the time of review, Equatorial Guinea and the Republic of Congo had no publicly available national AMR plans, highlighting persistent gaps in regional preparedness.^60^ Previous analyses indicate that the absence of a national plan is associated with weaker antimicrobial stewardship and limited policy coordination.^2,61^ Within the Economic Community of Central African States, high population mobility and cross-border trade further exacerbate the risk of transnational dissemination of resistant pathogens, reinforcing the need for harmonized regional governance frameworks.

These findings underscore that the presence of policy frameworks alone is insufficient; rather, the effectiveness of AMR governance in Central Africa is primarily determined by the degree of institutionalization, enforcement capacity and sustained financing. This review is therefore highly relevant, as it provides a structured regional synthesis of governance gaps, informing both national policymakers and regional bodies on priority areas for strengthening AMR control systems. Compared to the pre-2015 period, when AMR governance was largely absent or fragmented, with isolated initiatives confined to tertiary healthcare settings and minimal policy coordination, the current landscape reflects notable progress in the formal adoption of NAPs and alignment with the World Health Organization Global Action Plan. However, a decade later, this progress remains predominantly normative rather than operational, as implementation continues to be constrained by weak institutional anchoring, limited cross-sector integration under the One Health approach and persistent reliance on external funding. This contrast highlights a transition from policy absence to policy presence without full system functionality, underscoring the need to shift focus from strategic development to effective implementation and sustainability.

Even where NAPs exist, weak legal anchoring constrains implementation, accountability and cross-sectoral enforcement. Cameroon exemplifies this challenge, where policy intent is documented, but implementing instruments and clearly defined institutional responsibilities remain insufficiently specified. Evidence from other contexts suggests that NAPs require binding legal authority and integration into national regulatory systems to influence practice effectively.^62^ Weak legal mandates may also reduce stakeholder engagement and hinder the integration of AMR priorities into sectoral policies. Comparable governance limitations have been reported in the Democratic Republic of Congo and Gabon, where monitoring systems and accountability mechanisms remain incomplete. These findings align with broader evidence that fragmented institutional responsibilities and limited incentive structures undermine the sustainability of AMR governance.^63^

The operationalization of the One Health approach remains uneven across the subregion. Although several countries report multisectoral coordination platforms, their mandates, sectoral inclusiveness and functional continuity vary considerably. External partners frequently provide technical and financial support for establishing these platforms and implementing AMR-related activities.^64,65^ However, heavy reliance on donor funding introduces structural vulnerabilities, including limited national ownership, short implementation cycles and fragmented programme delivery.^66^ Governance domains such as participation, coordination, accountability and equity are consistently reflected in policy documents but remain insufficiently operationalized in routine practice.^67^

Importantly, compared with other African subregions, Central Africa appears to lag in operational integration of AMR governance. Countries in the West, East, South and North Africa have demonstrated more advanced implementation of integrated surveillance systems and more functional multisectoral coordination mechanisms.^68–71^ In contrast, Central Africa faces a higher burden of structural constraints, including institutional fragility, limited absorptive capacity and recurrent insecurity, which collectively impede sustained implementation. These differences suggest that AMR governance trajectories are strongly shaped by broader health system resilience and political stability.

Surveillance and monitoring systems represent a critical bottleneck in the region. Several countries lack fully operational national systems for AMR and antimicrobial consumption surveillance, particularly in the animal and environmental sectors. Deficiencies in laboratory accreditation, standardized protocols and digital infrastructure limit data comparability and delay the detection of resistance trends.^72^ Weak monitoring and evaluation systems further constrain adaptive governance, reducing the ability to generate feedback, inform policy adjustments and improve implementation over time.^73^

From a temporal perspective, the selection of the 2015–2025 period aligns with the global momentum generated by the World Health Organization Global Action Plan on AMR, which catalysed the development of national strategies worldwide. While this study does not systematically assess the pre-2015 landscape, available evidence suggests that AMR governance structures in Central Africa were largely fragmented or absent prior to this global policy shift. The findings of this review, therefore, likely reflect a phase of progressive but incomplete institutional development rather than fully mature governance systems.

Despite these challenges, the review identifies several actionable levers for strengthening AMR governance in Central Africa. The establishment of technical working groups with clearly defined mandates, investment in laboratory quality improvement systems and workforce capacity building represent feasible entry points for institutional strengthening. Incremental integration of AMR and antimicrobial consumption data into decision-making processes can enhance policy coherence and responsiveness. Strengthening domestic financing mechanisms is essential to ensure sustainability, as predictable funding supports routine coordination, procurement and laboratory operations. Legal harmonization across sectors may further improve regulatory enforcement and accountability.

Overall, these findings highlight that AMR governance in Central Africa is at a transitional stage, characterized by increasing policy alignment with global standards but limited operational maturity. Addressing the gap between policy commitment and implementation capacity should therefore be a central priority for advancing effective One Health governance in the region.

### Limitations of this study

An important limitation of this study is the uneven geographical distribution of available data, with a strong predominance of data from Cameroon. This imbalance limits the generalisability of the findings to the broader Central African subregion and reflects significant data gaps and limited documentation of AMR governance frameworks across several countries. The inclusion of grey literature, although necessary to capture policy documents and institutional reports, may introduce selection bias and variability in data quality, as such sources are not always subject to rigourous peer review.

Language bias may also have influenced the findings, as documents not available in English or French could have been underrepresented, potentially excluding relevant national or local evidence. Additionally, the heterogeneity of sources and the uneven availability of country-level data may have led to partial overgeneralization of findings across the subregion, particularly in contexts where empirical evidence remains scarce. Finally, although multiple Central African countries have adopted NAPs incorporating a One Health approach, this review was limited by the scarcity of empirical data on implementation outcomes, restricting the ability to fully assess the effectiveness and real-world impact of these governance frameworks.

### Strengths of the study

The strengths of this review support its contribution to the AMR governance literature in Central Africa. We synthesized empirical studies and national policy documents across eight countries within a consistent One Health framing. We also complemented database searches with structured retrieval of national documents through focal points. However, the evidence base remains uneven across countries, with documentation concentrated in Cameroon. Some findings rely on plans and reports that may reflect intended activities more than verified implementation. Grey literature availability and public access to national documents may also have influenced what could be retrieved and analysed. These data support cautious interpretation of cross-country comparisons, especially for implementation status.

### Conclusion

This research therefore highlights marked variation in AMR governance across Central Africa. Several countries, including Cameroon, Gabon, Chad, São Tomé and Príncipe, developed NAPs aligned with the WHO Global Action Plan. Equatorial Guinea and the Republic of Congo had no national AMR plan available at the time of review. These differences undermine regional coherence and limit routine alignment with international frameworks and standards, including GLASS-related guidance. Across countries with existing plans, implementation remained constrained by incomplete multisectoral coordination and weak regulatory anchoring. Sources also reported limited surveillance capacity and financing that was often partner-dependent and unpredictable. Despite these constraints, enabling practices were documented in some settings, including technical working groups with defined mandates and laboratory quality improvement initiatives. Strengthening AMR governance in Central Africa will require addressing persistent One Health implementation deficiencies through clearer and enforceable legal mandates, increased and predictable domestic financing and the operational integration of surveillance systems across human, animal and environmental sectors. Further research, including studies on antimicrobial consumption and on monitoring and evaluation of governance measures, is needed to support context-appropriate strategies and strengthen accountability.

## Supplementary Material

dlag093_Supplementary_Data

## Data Availability

No new data were generated or analysed in this study. All data supporting the finding of this systematic review are derived from previously published articles, which are cited within the manuscript.

## Supplementary data

Table S1 is available as Supplementary data at *JAC-AMR* Online.
