# Supplementary material for: Governance of antimicrobial resistance in Central Africa (2015–2025): a systematic review of policies, One Health coordination and funding
Source: JAC Antimicrob Resist. 2026 May 30;8(3):dlag093. doi: 10.1093/jacamr/dlag093 (PMC13221972; doi:10.1093/jacamr/dlag093)
Supplement: dlag093_Supplementary_Data [file dlag093_supplementary_data.docx]

**Supplementary Table 1:** Detailed overview of the articles included in the review.

| **Identifier /authors' names** | **Years** | **Country** | **Document type** | **Description** |
| --- | --- | --- | --- | --- |
| Trollip et al. | 2022 | Cameroon | Scientific article | Implementation of a customized antimicrobial resistance laboratory scorecard |
| Moffo et al. | 2021 | Cameroon | Scientific article | Challenges of integrating of antimicrobial resistance surveillance in food-producing animals and related public health risks in Cameroon |
| Vougat Ngom et al. | 2025 | Cameroon | Scientific article | Resistance to medically important antimicrobials in broiler and layer farms in Cameroon and its relation with biosecurity and antimicrobial use |
| Massongo et al. | 2021 | Cameroon | Scientific article | This study describes trends in antibacterial resistance at the National Reference Laboratory in Cameroon, comparing the situation between 2010 and 2017. |
| Yopa et al. | 2023 | Cameroon | Scientific article | Evaluation of the antimicrobial resistance surveillance system in sentinel sites in Cameroon. |
| Koudoum et al. | 2023 | Cameroon | Scientific article | Challenges in tackling antimicrobial resistance in resource-limited settings |
| Mouiche et al. | 2020 | Cameroon | Scientific article | Challenges of antimicrobial consumption surveillance in food-producing animals in Cameroon from 2014 to 2019 |
| Acho and Toby | 2023 | Cameroon | Activity report | Strengthening multisectoral coordination to contain antimicrobial resistance in Cameroon |
| Mouiche et al. | 2019 | Cameroon | Scientific article | Antimicrobial resistance from a one health perspective in Cameroon |
| Ngaruka et al. | 2021 | D.R. Congo | Scientific article | This study describes the consumption habits of animal-derived foods among outpatients diagnosed with antimicrobial resistance in Bukavu, Democratic Republic of Congo |
| Adegbite et al. | 2022 | Gabon | Scientific article | Healthcare providers’ awareness and prescribing practices on antimicrobial resistance in Lambaréné, Gabon. |
| Akilimali et al. | 2022 | D.R. Congo | Scientific article | The study examines patterns of antimicrobial consumption and resistance in the Democratic Republic of the Congo and provides policy-oriented recommendations. |
| Bunduki et al. | 2019 | D.R. Congo | Scientific article | Antimicrobial resistance challenges and lessons learned from healthcare practices in conflict-affected Eastern Democratic Republic of Congo. |
| Akilimali et al. | 2023 | D.R. Congo | Scientific article | Current status and strategic perspectives on antimicrobial resistance in the Democratic Republic of Congo |
| Njukeng et al. | 2019 | Cameroon | Scientific article | Confronting antimicrobial resistance in Cameroon |
| Osena et al. | 2025 | Cameroon, Gabon | Scientific article | Gaps in testing practices and inadequate digital infrastructure are causing high variability in AMR in Africa |
| Djuikoue et al. | 2022 | Cameroon | Scientific article | Practices relative to antimicrobial resistance |
| Vounba et al. | 2022 | Gabon | Scientific article | Microbiology laboratories involved in disease and antimicrobial resistance surveillance |
| Kouomogne-Nteungue et al. | 2024 | Cameroon | Scientific article | Influence of infection prevention and control in disease transmission in healthcare |
| RDC- NAP-AMR | 2018 | D.R. Congo | Policy Document | National plan to combat antimicrobial resistance |
| RCA- NAP-AMR | 2023 | Central African Republic | Policy Document | National multisectoral action plan to combat antimicrobial resistance in the Central African Republic 2024–2028 |
| Chad- NAP-AMR | 2024 | Chad | Policy Document | National strategic plan to combat antibiotic resistance 2025–2028 |
| CMR- NAP-AMR | 2024 | Cameroon | Policy Document | National action plan on antimicrobial resistance 2024–2028. |
| Gabon- NAP-AMR | 2025 | Gabon | Policy Document | National plan to combat antimicrobial resistance 2025 – 2029 |
| CMR- NAP-AMR | 2018 | Cameroon | Policy Document | 2021-2023 action plan for the responsible management of antimicrobials in Cameroon |
| ST&P- NAP-AMR | 2023 | Sao Tome &  Principe | Policy Document | National plan to combat antimicrobial resistance NAP 2023–2025 |
